# Giant Cell Tumor of Rib Arising Anteriorly as a Large Inframammary Mass: A Case Report and Review of the Literature

**DOI:** 10.1155/2012/850509

**Published:** 2012-11-27

**Authors:** Amit Sharma, Amy E. Armstrong

**Affiliations:** ^1^Division of Transplantation Surgery, Department of Surgery, Virginia Commonwealth University, P.O. Box 980057, Richmond, VA 23298, USA; ^2^Virginia Commonwealth University School of Medicine, 1101 E. Marshall Street, Richmond, VA 23298, USA

## Abstract

*Introduction*. Giant cell tumor of the bone is a rare benign lesion that infrequently affects the ribs, and if present, is usually located posteriorly. The rarity of this tumor poses diagnostic and therapeutic problems for physicians, especially when it is located in the anterior arc of the rib in close proximity to the breasts in female patients. *Case Presentation*. We report the case of a 32-year-old Asian female with a giant cell tumor of her anterior rib, presenting as a large inframammary mass. Computed tomography showed a tumor arising from the 7th rib anteriorly with marginal sclerosis, cortical destruction, and a soft tissue mass. She was treated with surgical resection, and the defect was reconstructed primarily. The surgical specimen measured 28.0 × 24.0 cm. The microscopic examination showed a large number of multinucleate giant cells scattered over the parenchyma. Patient recovered uneventfully and continues to be recurrence-free six years after surgical resection. *Conclusion*. We report the largest known case of giant cell tumor arising from the anterior aspect of a rib. We recommend including giant cell tumor in the differential diagnosis of chest wall masses especially in female patients, regardless of the size on clinical examination.

## 1. Introduction

Giant cell tumors (GCTs) of bone usually arise in the epiphyseal region of the limbs, and their occurrence in the ribs is unusual [1]. These are usually found posteriorly in the ribs (epiphysis of head and tubercle), and their presentation anteriorly is very rare [2]. Giant cell tumors of the anterior rib in close proximity to the breasts may pose diagnostic and therapeutic problems [3], which prompted us to report this unusual case. 

## 2. Case Presentation

**Figure 1:**
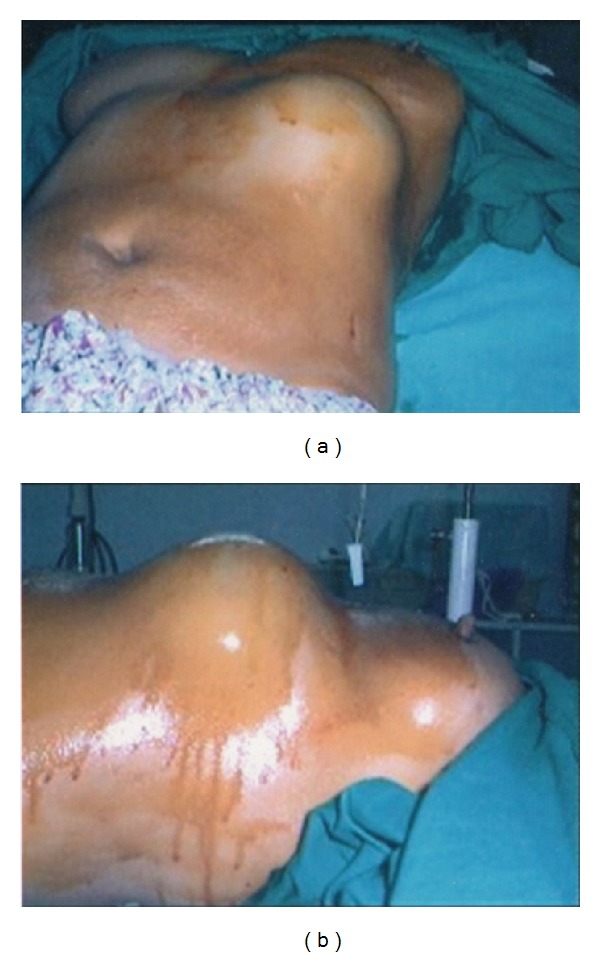
Clinical appearance of the left inframammary giant cell tumor. Picture shows the left inframammary mass just prior to surgical resection (after prepping with betadine solution) in frontal (a) and lateral (b) view.

MK, a 32-year-old Asian female, was admitted to our surgical unit with the chief complaint of a slowly enlarging mass just below her left breast for past 4 years. The only associated symptom was mild occasional pain of recent onset. Clinically, the lump was approximately 20.0 × 15.0 cm, hard in consistency, mildly tender, and located anteriorly below the left inframammary fold (Figure 1). The margins were indistinct, and the surface was smooth with tense, patchily erythematous overlying skin. 

**Figure 2:**
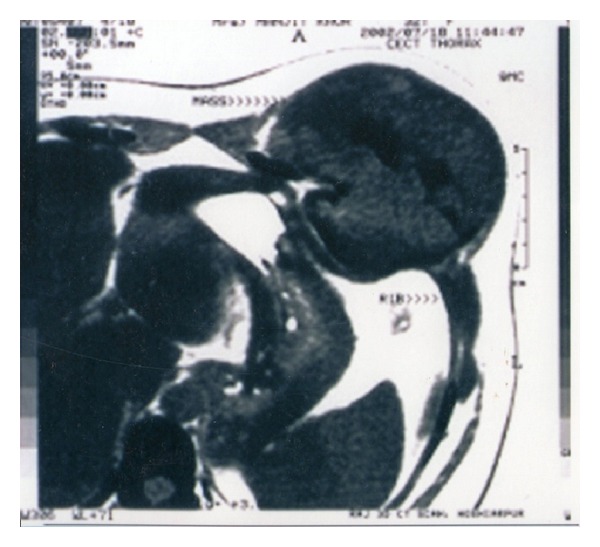
Giant cell tumor on CT scan of the chest. Computed tomography of the chest showing a large soft tissue mass arising anteriorly from the 7th rib, causing cortical destruction.

Chest and abdominal radiographs showed a radiolucent shadow in the left lower chest region. Computed tomography of the chest (Figure 2) showed a tumor arising from the 7th rib anteriorly with marginal sclerosis, cortical destruction, and a soft tissue mass. The patient was investigated to rule out hyperparathyroidism. 

**Figure 3:**
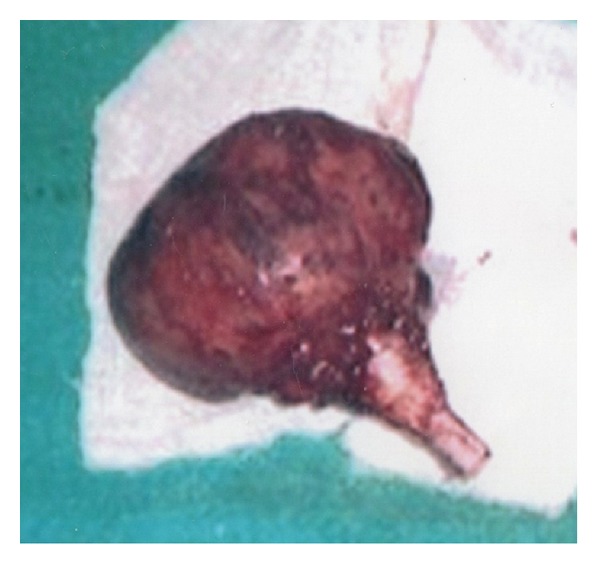
Gross specimen after surgical resection. Specimen showing giant cell tumor measuring 28.0 × 24.0 cm with an excised portion of the 7th rib.

The tumor was excised along with the rib above and below and a length well past the tumor margin. The defect was reconstructed primarily, and the patient made an uneventful recovery. Histopathology reported a large lobular mass enclosing the rib and measuring 28.0 × 24.0 cm (Figure 3). Cut section showed areas of necrosis, hemorrhage, and a gritty feel. 

**Figure 4:**
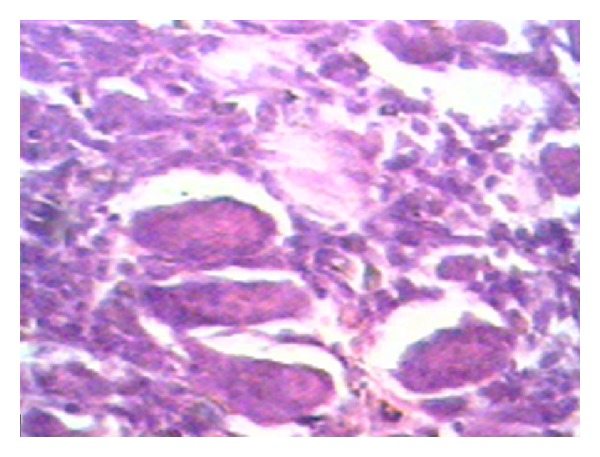
Histopathology of giant cell tumor. Microscopic examination with hematoxylin and eosin staining showing large number of multinucleate giant cells scattered over parenchyma with spindle-shaped mononuclear stromal cells (magnification ×100).

Microscopic examination revealed a large number of multinucleate giant cells scattered over the parenchyma (Figure 4). The stroma contained vesicular plump spindle cells with nuclei. There were large areas of hemorrhage and necrosis. A final impression was made of a grade III giant cell tumor of the rib. The patient remains recurrence free 6 years after tumor excision.

## 3. Discussion

Giant cell tumors of the bone account for 5% of all primary bone tumors [4]. Most (85%) occur in the long bones, and approximately 50% are found around the knee joint. Many large series have reported an incidence of around 1% in the ribs; after reviewing 15 cases, Gupta and Mittal show that most of these involved the posterior aspect of the rib [2]. Microscopically, the two basic components of benign GCTs are stroma and multinucleated giant cells; the stromal cells are mononuclear and may be spindle shaped, ovoid, or round, while the multinucleated giant cells may be so large that the numerous nuclei are almost uncountable. The frequency of multinucleated giant cells is variable and most likely dependent on stromal pattern [5]. Variants of GCTs include chondroblastoma, chondromyxoid fibroma, aneurysmal bone cyst, and “brown” tumor of hyperparathyroidism [6]. When differentiating GCTs of rib from simple bone cyst Oschner described that the latter are more likely to be formed in the anterior part of the ribs, whereas GCT are mostly located posteriorly in the epiphysis of bone (i.e., the head and tubercle of ribs) [7]. Only 3% of GCTs develops in the immature skeletons which distinguishes these patients from those with aneurysmal bone cysts, in whom the tumor maximally occurs prior to epiphyseal fusion [6]. 

Giant cell tumors are aggressive tumors and present with the signs and symptoms of pain, swelling, and limitation of motion about a joint. Hutter et al. report that patients experienced symptoms for an average of 10 months prior to first treatment of benign GCT [5]. However, our patient noted a slowly growing inframammary mass over a period of 3 to 4 years, and the occasional pain began near her time of presentation. This delayed presentation probably contributed to the extremely large tumor size found at the time of surgical resection. While multiple cases of GCT originating from the rib have been reported, the two-dimensional span of 28.0 × 24.0 cm resected in our patient appears to be the largest to date (Table 1) [4, 8–14]. 

Current methods available to treat GCT include curettage with or without the use of alcohol, liquid nitrogen, phenol or methylmacrylate or bone graft, and complete surgical resection of the affected segment of bone [8]. Excision is desirable as 10% of GCTs in ribs undergo malignant transformation [7], while radiation therapy is not recommended as most of malignant transformations are related to previous radiation therapy [4]. Thus en bloc excision is an appropriate treatment, and disease-free survival is directly proportional to negative resection margin [15]. Hutter et al. report that most recurrences (81%) appear in less than 2 years, and almost all have been manifested by 4 years. Thus at least 5 years of close followup are recommended. However, it has been reported that the course of a benign giant cell tumor undergoing malignant transformation may take longer than 5 years [5]. 

## 4. Conclusion

In conclusion, ribs are a rare site for giant cell tumor and when present most of these tumors are located posteriorly near the epiphysis of the rib. We report the largest known case of giant cell tumor located on the anterior aspect of rib that was successfully managed with wide excision and primary repair of the chest wall defect without any recurrence. 

## Figures and Tables

**Table 1:** Published cases of giant cell tumors arising from anterior arc of the rib.

Author	Location	Surgical specimen size (cm)^a^
Riddle et al. [8]	5th anterior	5.0 × 5.0 × 4.5
Sakao et al. [9]	5th (anterior?)	5.8 × 5.2
Sakao et al. [9]	2nd anterior	6.0 × 3.5
Tavecchio et al. [10]	11th (entire floating rib)	7.0 × 6.0
Shin et al. [11]	2nd anterior	8.0 × 6.5 × 6.0
Sakao et al. [9]	2nd anterior	9.0 × 7.0 × 5.0
Al-Otaibi et al. [12]	9th anterior	9.5 × 6.5 × 3.0
Sakao et al. [9]	4th anterior	10.0 × 7.0 × 5.0
Sakao et al. [9]	3rd anterior	11.0 × 12.0 × 13.0
Dehghan et al. [4]	4th anterior	12.5 × 10.5 × 5.7
Briccoli et al. [13]	9th anterior/posterior	13.0 × 11.0 × 2.5
Sakao et al. [9]	4th anterior	15.0 × 7.5 × 5.5
Cordeiro et al. [14]	4th and 5th anterior	25.0 × 17.0

^a^In our case report the giant cell tumor originated from the anterior aspect of the left 7th rib. The excised specimen measured 28.0 cm × 24.0 cm.
